# Biological Evidence Management for DNA Analysis in Cases of Sexual Assault

**DOI:** 10.1155/2015/365674

**Published:** 2015-10-26

**Authors:** Teresa Magalhães, Ricardo Jorge Dinis-Oliveira, Benedita Silva, Francisco Corte-Real, Duarte Nuno Vieira

**Affiliations:** ^1^Department of Legal Medicine and Forensic Sciences, Faculty of Medicine, University of Porto, Porto, Portugal; ^2^Forensic Sciences Center (CENCIFOR), Coimbra, Portugal; ^3^Institute of Research and Advanced Training in Health Sciences and Technologies (IINFACTS), Department of Sciences, University Institute of Health Sciences (IUCS), CESPU, Gandra, Portugal; ^4^UCIBIO/REQUIMTE, Laboratory of Toxicology, Department of Biological Sciences, Faculty of Pharmacy, University of Porto, Porto, Portugal; ^5^Faculty of Medicine, University of Coimbra, Coimbra, Portugal

## Abstract

Biological evidence with forensic interest may be found in several cases of assault, being particularly relevant if sexually related. Sexual assault cases are characterized by low rates of disclosure, reporting, prosecution, and conviction. Biological evidence is sometimes the only way to prove the occurrence of sexual contact and to identify the perpetrator. The major focus of this review is to propose practical approaches and guidelines to help health, forensic, and law enforcement professionals to deal with biological evidence for DNA analysis. Attention should be devoted to avoiding contamination, degradation, and loss of biological evidence, as well as respecting specific measures to properly handle evidence (i.e., selection, collection, packing, sealing, labeling, storage, preservation, transport, and guarantee of the chain custody). Biological evidence must be carefully managed since the relevance of any finding in Forensic Genetics is determined, in the first instance, by the integrity and quantity of the samples submitted for analysis.

## 1. Introduction

Biological evidence with forensic interest may be found in several cases of assault, being particularly relevant for sexually related ones. Sexual aggression constitutes a serious social and public health problem that calls for an urgent forensic medical examination (FME), particularly in acute cases, that is, when the elapsed time between the assault and the FME is less than 72 hours, in the generality of cases [1–6].

In these cases a large number of forensic areas are involved (e.g., clinical forensic medicine, genetics, and toxicology) aiming to obtain the proof and elaboration of a final forensic report [1].

From the forensic intervention perspective, despite some published protocols and guidelines, few countries have officially adopted guidelines for evidence management, namely, in acute sexual assault (ASA) cases. Even when guidelines are adopted they may vary within the same country, between different regions and different institutions. However, to standardize the FME of ASA victims and the credibility of forensic practices, which are essential during judicial proceedings, clear guidelines developed by the scientific community are required [2, 6]. These guidelines will aid in optimizing forensic intervention and reduce unnecessary variations in the procedures, as well as improving collaboration among several entities and professionals, while enabling a well-timed and comprehensive forensic evaluation. An essential part of these guidelines should concern management of biological evidence for DNA analytical studies.

This work will focus on the management of forensic evidence, more specifically the biological samples. Indeed, examiners performing FME in ASA cases must have knowledge and training in collecting and handling evidence, always respecting guidelines and legal obligations. This is true regardless of the value of other forensic procedures (e.g., forensic interview, forensic medical history, photo documentation, or physical examination) that may be required. Examiners should also be aware of the scope and limitations of laboratory analysis as well as the consequences of contamination or degradation of any evidence [7]. Moreover, the interpretation of the findings related to evidence should also receive careful and thorough consideration, as there are multiple variables that may influence the quality of evidence [8, 9]. All these variables should be taken into consideration and discussed in any recommendations or guidelines, as well as in the expert (medical or laboratorial) reports.

Because of its utility in proving the occurrence of sexual contact and the identification of the suspects, biological evidence for DNA studies is nowadays considered the most important evidence for legal proof in courts of law [4, 10–12]. The proper handling procedures during selection, collection, packaging, labeling, storing, and transportation of evidence to the laboratory are key steps aiming to achieve final valid and reliable results [8, 9]. Oversights or faults in these procedures can call into question the production of the proof, namely, regarding evidence preservation (loss or contamination) and chain of custody [13].

In this study, we aimed to review and update forensic procedures already implemented in various forensic institutions. These are based on Portuguese and international forensic expertise and evidence gathered through the review of scientific literature and institutional guidelines. However, it is important to note that the application of these guidelines is highly dependent on the available local resources and should be mainly regarded to promote the quality and safety of forensic practices and fill some existing gaps.

It is hoped that this work can be a useful tool (not only for forensic practitioners) to help the mission of forensic expertise regarding ASA, promoting the ability of professionals to detect, collect, and properly appraise biological forensic evidence.

## 2. Forensic Evidence

In every crime against people, as in sexual assault, the contact between the perpetrator and the victim, or his/her environment, or both always leaves evidence which is transferred from the perpetrator to the victim, to the scene, and vice versa [8, 14, 15].

Forensic evidence, in the broadest sense, is any item or information about a suspected crime, which is considered to be relevant to an investigation in order to find the truth of the facts. It may be useful to (1) orient police investigation; (2) provide a reliable identification of the perpetrator; (3) exonerate a suspect or an accused from a crime; (4) support or contradict a victim's, witness', or suspect's statement and, consequently, promote police to conduct further investigations; (5) provide information about the crime scene; and (6) provide proof that attests to the occurrence of the alleged event.

Typically crime scene evidence could be found on any place where a criminal offence was committed, on anything worn or carried by the victim during the time the offence or within or on the body of any person associated with the offence.

Evidence may be found at the victim's body or clothes, in condoms or bed clothes, or at the crime scene [2, 11, 16–19]. Therefore, the examiner should rapidly inform police to isolate and protect the crime scene and should collect first the more urgent samples [20]. Nevertheless, professionals must be aware that in ASA cases the victim's body may be the most important part of the crime scene [5].

Two types of evidence can be considered:

- Direct evidence: it establishes the fact without needing further investigations. The most important one is the eyewitness or victim statement; nevertheless, their statement can be prone to many inaccuracies and may be contradicted or supported by other types of evidence (e.g., biological evidence for DNA testing) [6].
- Circumstantial evidence (or indirect evidence): it needs to be identified and matched with a control or reference sample collected from the victim, suspect, and/or the crime scene or database. Although it is more objective than direct evidence, it must be handled carefully aiming to avoid risk of destruction, contamination, or loss. It is the majority of the evidence analyzed in the forensic laboratories and can be divided into two basic classes. (1) Physical evidence: it includes items of nonbiological origin, such as finger and foot prints, shoe/tire impressions, fibers, paint, soil, dirt, glass, headlamps or arson debris, explosives and gunshot residues, and figured injuries (e.g., bite marks, scratches) [6, 21]. They are very useful to identify the crime scene and should be collected when available [8, 14, 22]. (2) Biological evidence: it includes items from a biological origin, usually from the victim or the perpetrator (e.g., semen, vaginal fluid, oral fluid, sweat, blood, and other body fluids, hair, cells of the alleged perpetrator under a victim's finger nails, or epithelial cells of the alleged victim present on the penis of the perpetrator) [19, 23], and botanical elements (e.g., pollen, plants, and wood). It is considered the most important type of evidence (especially semen) since it is very useful to prove that physical/sexual contact occurred and to identify a perpetrator through DNA studies [5, 9–11, 24].

## 3. Biological Evidence

The collection of biological evidence for DNA studies is particularly useful in ASA cases to establish the occurrence of sexual contact and to proceed with suspect identification. In fact, the presence of semen on a prepubertal child's body, clothes, or vicinity during the FME usually confirms the diagnosis of the sexual contact and is generally accepted in a court of law as proof [3, 17–20, 25, 26]. Nevertheless, this interpretation should not be regarded as an irrefutable proof, especially for incest or intrafamily cases, since a secondary transfer of sperm cells from adult clothing/bedsheets to babies' or children's clothing during laundry washes was previously evidenced [27, 28]. Moreover, a complete genetic profile of the father can be obtained despite the fact there was absolutely no sexual abuse involved [27, 28].

The following considerations should be taken in order to help forensic examiner to define the best practices in each case and to interpret the findings:

- Semen (spermatozoa suspension in the seminal fluid): it is rarely present in oral, anorectal, and vaginal cavities 6, 24, and 72 hours after sexual contact, respectively [22, 29]. In vaginal cavity the half-life time depends on the age of the victim (pre- or postpubertal) and if the semen is localized in the cervix, the half-life may be much higher than 72 hours [22, 29]. In postpubertal girls spermatozoa may remain motile in the vaginal secretions for 6 to 12 hours and in the cervix for as long as 5 days [30]; nonmotile spermatozoa may be found in stains of vaginal secretions from 12 to 48 hours after ejaculation [22, 29]. The half-life of semen in the prepubertal girls is comparatively shorter due to the absence of cervical mucus [22, 29]. Dried secretions on clothing remain quite stable, so that semen may be detected for longer than 1 year [22, 31]. These half-lives represent mere estimations, since several variables (that should be described in the forensic medical report) must be considered when documenting the presence or not of semen in sexual assault cases [22, 29, 31]: (1) the type of practice and circumstances (e.g., where evidence is deposited; ejaculation occurred in the skin, oral, anal, or vaginal mucosa or in the cervix; condom use; the perpetrator is azoospermic or vasectomized); (2) the time between sexual contact and evidence collection; (3) victim's gender, age, and activities (e.g., urinating, defecating, vomiting, brushing teeth, bathing, eating, drinking, smoking, spitting, running, and walking) after sexual contact.
- Observation of spermatozoa under an optical microscope (e.g., using stains such as the Kernechtrot Picroindigocarmine (KPIC; or Christmas Tree stain), Giemsa, hematoxylin/eosin, and methylene blue/eosin) or by phase contrast microscopy (no stains): these are considered for diagnosis of sexual contact and the concomitant observation of motile spermatozoa allows estimating the time of the assault. However, since these techniques do not lead to the identification of the perpetrator and biological material is lost to perform smears, some authors do not recommend this procedure. The absence of spermatozoa may occur if the suspects are azoospermic or vasectomized or if semen stains are dry [24, 32]. Under an optical microscope, the Florence Iodine (FI) test is used for seminal fluid identification by detecting the presence of choline through the addition of an iodine based reagent, which produces characteristic brown choline periodide crystals. In a recent study, Hardinge and colleagues [33] observed that prostate-specific antigen (PSA) is much more sensitive but less specific than the FI test to confirm the presence of seminal fluid.
- Seminal acid phosphatase (AP): this enzyme is present in semen and for positivity, the presence of spermatozoa is not needed since it is a prostatic enzyme. In postpubertal girls' vagina or cervix, the possibility to register elevated AP levels ranges from 24 hours [24] to 72 hours after ejaculation [22, 29]. AP levels are elevated for a much shorter time in mouth (perhaps only 6 hours) and in the rectum (less than 24 hours), but only estimates are available [34]. On the other hand, in spite of an elevated level of AP being a specific indicator of recent sexual intercourse and ejaculation, its use as evidence is somewhat limited due to the existence of an isoenzyme in low levels in postpubertal vaginal fluid and female urine [22]. The presence and concentration of AP in prepubertal girls is unknown. Analytical techniques to quantify AP (e.g., Brentamine Fast Blue reaction) should be regarded as guide and if result is negative, DNA studies (autosomal STRs and Y-STRs) must proceed [35]. Indeed, the results of the Brentamine colorimetric reaction may be difficult to interpret due to the interference of fabric colors and therefore may lead to false negative results.
- Prostate-specific antigen (PSA): it is a serine protease produced by prostatic epithelial cells found in many tissues (e.g., seminal fluid, prostatic fluid, male serum, male urine, apocrine sweat glands, and breast milk from lactating women). Although PSA is not tissue and gender specific, in ASA cases, the interpretation of the results should not pose a significant problem due to its low concentrations in nonprostatic fluids [36–38]. PSA can be found up to 48 hours in postpubertal girls' vagina or cervix [24]. PSA is considered one of the most sensitive methods for semen detection and can be applied for azoospermic individuals. Similar to AP, PSA should be regarded as guide and if result is negative, DNA studies (autosomal STRs and Y-STRs) must be performed.

Other aspects should be considered:

- Oral fluid: it constitutes the second biological evidence commonly found in ASA cases, often observed by the Phadebas test, which detects *α*-amylase activity. Nevertheless, it should be taken into account that *α*-amylase can be present in body fluids other than saliva. This biological material transports epithelial cells from buccal mucosa which contains DNA [3, 4, 23, 39, 40]. It is very useful since the perpetrator commonly licks, bites, or kisses the victim, and his/her oral fluid may prevail on the victim's skin (e.g., neck, thorax, and abdomen). Cigarette filters, bottles, or cans of soft drinks are likely to lead to the identification of the perpetrator. Stamps and envelopes are less likely to provide DNA that could lead to a perpetrator because they are usually now self-adhesive and therefore few people lick them anymore.
- Some studies argue that perpetrator's DNA may be detected in the victim's oral cavity up to 1 hour after intense kissing [41]. Nevertheless, collection within this period is very difficult to accomplish, since the victims are presented later for FME and usually wash their mouth. Thus the collection of oral fluid must be performed as soon as possible for victim's hygiene and comfort but also for avoiding loss or destruction of this sensitive evidence that normally presents low amounts of DNA.
- Head or pubic hair, and/or epithelial cells of the victim or perpetrator transferred between them during the sexual contact or a fight, should also be collected with utmost care due to the low amount of DNA present [2, 4, 5, 42–46]. It should be born in mind that the pubic hair transferred during intercourse, victim being in the dorsal decubitus position, is minimal even if samples are collected during a short time afterwards, as previously demonstrated [45].
- The fingernail hyponychium is an isolated area where evidence may accumulate and can provide a valuable source of evidential material for investigation. During the course of a sexual assault, trace amounts of skin (especially if the victim scratched the perpetrator), body fluids, hairs, fibers, and vegetation may collect under the nails of either the victim or perpetrator [42, 43, 47]. The persistence of foreign DNA did not tend to last beyond 6 h [42].

## 4. Evidence Preservation

Evidence preservation aims to avoid its destruction, contamination, or loss.

### 4.1. Destruction

To avoid the destruction of evidence, the professional to whom the case was reported should inform the victim or any person who reported the incidence/offence about practices that the victim should refrain from until FME can be completed [3–5, 26, 48, 49]:

- shower or wash any part of the body, including mouth, hands, and head hair;
- brush teeth;
- clean or cut fingernails;
- comb or cut paint hair;
- perform vaginal irrigation;
- urinate, defecate, or vomit (and if this is imperative, do it in a clean container with a lid);
- eat, drink, chew, or smoke;
- run or perform any kind of sport activities or the same;
- change, wash, or destroy clothing worn during the event;
- change or destroy sanitary pads worn during the event;
- touch the crime scene (including emptying garbage can or flushing the toilet).

Moreover in order to prevent DNA degradation, the forensic examiner must correctly select the type of material used for collection and storage (e.g., paper versus plastic containers—please see Section 5.3) and ensure complete drying of the sample prior to packaging [9, 13, 50].

### 4.2. Contamination

For DNA studies, one of the greatest laboratory barriers is the contamination of genetic material from other sources (e.g., from the examiner and other biological evidence). Contamination may occur during the sexual contact (e.g., if there is more than one perpetrator), during the period between the sexual contact and the FME, during the FME, and in the laboratory [51–53]. In order to avoid it, examiners should take special precautions to prevent cross-contamination between evidences [7, 29, 50]. For this purpose, it is important [4, 54]

- to work under aseptic conditions to avoid microbial contamination;
- to always use disposable supplies to ensure individual protection (e.g., gowns, powder-free gloves, mask, or other protective clothing) and to avoid direct contact with the samples;
- to ensure that the room where FME takes place is regularly cleaned before and after patient use;
- to avoid sneezing, coughing, or talking over the samples;
- not to drink, eat, and/or smoke when handling samples;
- to store swabs or other samples separately, ensuring that they are contact-free (e.g., while drying or during storage), particularly in circumstances where reference samples were also collected at the same time [49];
- to avoid contamination with evidence of other body areas, since the specific location of each biological sample is crucial to the investigation [29]; gloves should be changed regularly between the collections of each item of evidence;
- not to touch the cotton-tipped swabs.

### 4.3. Loss

In many ASA cases, the evidence is recovered in very low amounts. Consequently, two issues must be weighed: the number of swabs to be performed during the collection for each evidence and the pertinence (or not) of doing semen smears for spermatozoa observation under optical microscope.

The number of swabs performed per body area is important for financial reasons but also due to evidence concentration in each swab. Hochmeister and Ferrel [49] consider that one swab per item of evidence is more than enough. Others advise to collect at least two swabs for the same item of evidence [22, 29, 55, 56]. The medical examiner should justify the adopted procedure in the FME report and consider the following objectives in the decision making process: (1) ability to conduct independent analysis for counterproof; (2) collection of all biological evidence available; and (3) facility to collect evidence. Therefore, it is important to highlight how each technique meets these objectives [6]:

- One swab: it is a rapid technique but does not guarantee that the entire evidence is collected for laboratory analysis. It is particularly useful in the presence of evidence with limited quantity. Moreover, if counterproof is required, two situations may be possible: (1) half the cotton swab could be preserved in the laboratory allowing one to perform a new analysis that will begin from extraction of DNA; (2) the entire swab is used in the first forensic analysis (most common situation) and counterproof analysis must be made from DNA previously extracted, ensuring that both analyses begin from the same DNA sample.
- Two swabs simultaneously: in this case, biological material will be divided into two swabs, which could reduce the success of the laboratory analysis. Furthermore, nothing can guarantee that the two swabs, even used together, have the same evidence quantity, which for some authors seems to be relevant for legal issues. The evidence is rapidly collected and allows the use of the second swab for counterproof [29, 55, 56]. In anogenital area this technique is only performed in adult or postpubertal victims. It should be considered when there is enough biological material available (e.g., direct ejaculation within vaginal cavity occurred).
- Two swabs successively or “double swab technique”: it is the application of two successive swabs, the first being wet (aiming to collect the majority of the evidence) and the second being a dry swab passed through the same place, the order of the swabs being annotated. This technique aims to collect the largest quantity of evidence available. It is not rapid and there is no guarantee of equality of the two swabs (the second swab may have much lower concentration of the evidence), reducing usefulness in counterproof. In spite of these limitations, this technique has been widely reported in the literature for the collection of various different biological samples (e.g., perpetrator's oral fluid or epithelial cells on the victim's skin) due to good outcomes [23, 39, 47].

In the majority of cases, semen smears for spermatozoa observation under optical microscope should not be performed, except in very specific circumstances, which should be detailed in the FME report. The following reasons justify their uselessness [49]:

- Many variables impact the semen motility and its observation by the examiner does not give a precise estimation of the time of the sexual contact.
- Due to the increasing number of vasectomized individuals, a semen smear has become a less effective screening tool to prove sexual contact.
- DNA analysis will be performed in the forensic laboratory regardless of the examiner's findings on a smear.
- Precious DNA evidence studies may be wasted by preparing a smear.

## 5. Evidence Management

Good evidence management must properly ensure procedures in the sequence ranging between selecting and collection, packing, sealing, labeling, and insertion into the* kit*, its storage, preservation and transportation, and reception by the forensic laboratory, always ensuring the compliance of chain of custody.

### 5.1. Evidence Selection

The details of the sexual assault history and the physical exam should guide the examiner for evidence collection [3, 4]. During the physical examination, an alternate light source may assist the detection of some findings (that may need special techniques for visualization such as injuries) which may be invisible to the naked eye [57, 58]. Lamps are also an effective alternative to chemical-based screening tests.

Semen is very fluorescent in nature and the fluorescence can be observed on dark as well as light textiles when illuminated with an intense UV light, without the need for using colored goggles. To detect semen the standard Wood's lamp (wavelength 360 nm), often used during SAS examinations, has been shown to be ineffective since several creams and ointments fluoresce in a similar manner to semen [59]. Instead, other light sources, with appropriate filters [59], may be used with the understanding that relatively fresh semen might be more easily observed with the naked eye than with an alternative light source [29]. Application has been possible on skin surfaces and vaginal, anal, and pharyngeal mucosa. The Polilight has also been considered a useful light source to detect biological samples such as semen, oral fluid, and bloodstains (e.g., on clothing) [60–62].

### 5.2. Evidence and Reference Sample Collection

In ASA cases, biological fluids collected on cotton-tipped swabs are the most important items of trace evidence. However, all evidence must be collected since, in most cases, it is not possible to collect it later on; even if evidence is still intact, the chain of custody may already be “broken” and evidence will be compromised and therefore should not be analyzed since it will not be admissible in a court of law. For this reason, it is advisable to collect any evidence relevant to the case even though only some samples may be subjected to laboratory analysis [7, 50].

The technique and materials used to collect evidence depend on the type of evidence and its support. For DNA analysis, swabs are usually preferred to collect semen and other fluids, but different techniques exist for hair collection, for example. The presence of inhibitors is another limitation that sometimes examiners have to face [16]. Indeed, substances such as indigo dye present in denim affect the PCR amplification and therefore compromise the DNA results [63, 64].

#### 5.2.1. Swab Techniques

Depending on the purpose, swabs of different design, shape, and size are commercially available and should be judiciously selected. Synthetic swabs (e.g., flocked nylon) are now available and are proved to be more efficient at releasing cells during the extraction [26] than cotton-tipped ones. Generally, the collection should be done by gently (to prevent exfoliation of the victim's own epithelial cells) rubbing in a circular motion for 15 seconds, a restricted area of the mucosa or skin, from the periphery to the center and rotating the swab. In the following, specific collection procedures are briefly outlined according to surface type examined [3, 46]:

- For dry surfaces (e.g., skin) the swabs should be slightly moistened with 1-2 drops of sterile distilled water, “damp swab.” Phosphate buffered saline (PBS) is also advocated (e.g., penile swab) since it prevents cells rupturing or shriveling up due to osmosis. Therefore, visualization of spermatozoa or vaginal epithelial cells from swabs is more prone to be successful, especially if the number is reduced. PBS does not affect subsequent DNA analysis. The same is not true for certain saline solutions and tap water due to electrolytes content and pH [65].
- For mucosa/epithelium (e.g., oral) or other wet surfaces, a dry swab should be used.
- To collect evidence from underneath fingernails, a damp, small, and thin tip swab should be used to be able to reach under the fingernails.

All collected swabs should be air-dried at room temperature for a minimum of one hour [22]. To accelerate drying, a cold hair-dryer (not heat to dry swabs) or a swab dryer may be used [22] and the chosen procedure should be described in the FME report. Nevertheless, it should be highlighted that the use of a cold hair-dryer to dry swabs is controversy since it could promote cross-contamination of samples by having biological material fly inside the hood or over the area where swabs are being dried and on the hair-dryer itself. Some of this material could end up on subsequent swabs to be dried. Therefore it is mandatory that each swab is dried separately and the hood/area/dryer must be thoroughly cleaned between samples to prevent contamination.

#### 5.2.2. Other Techniques

Other techniques to collect biological evidence (e.g., loose hairs) or physical evidence may be used, such as disposable plastic tweezers, combs, or scrapers (the latter for fingernail evidence). All specimens should be collected separately and packaged inside a little paper bag or in a bindle (piece paper folded in order to hold evidence at the center, avoiding loss in the folds of the envelope or bag; the sheet is folded in half and then folded again into three equal parts).

In case of stains on substrates that can be brought in the laboratory (e.g., clothes), collection should be performed in the laboratory and not on-site. Clothes or other items should be collected separately in appropriate paper bags [3].

Disposable plastic pipettes may be useful to collect liquid remains and, in this case, the material must be packed in a tube or another suitable container.

Tape adapted to evidence collection (e.g., on clothing or other support) could be repetitively applied at the suspected sites and then placed directly in the DNA extraction tube [66].

If a broken fingernail is collected as evidence, the cut should be performed away from the broken area.

#### 5.2.3. Reference

Reference biological sample from the suspect and the victim should be collected to perform comparative DNA testing and then correctly labeled to avoid confusion with the evidence [67]. An oral fluid reference sample is usually performed using a foam swab [4]. Most frequently, appropriate reference swabs (e.g., serrated) are used to vigorously rub the oral mucosa (inner cheek) in order to collect some mucosal cells, as rapidly and painlessly as possible.

If oral-genital contact is suspected, blood or hair sample may be preferred to act as reference sample since oral sample might be contaminated with the perpetrator's DNA. A blood sample should be collected by venipuncture and deposited onto an appropriate support (e.g., clean white cotton fabric, cellulose, and FTA paper (promotes long term storage of the blood/DNA at room temperature)). If it is suspected that a blood transfusion or bone marrow transplantation has been performed, the blood sample is not advisable and a hair or oral sample should be collected instead [68]. To collect a hair sample, at least 7 hairs should be pulled out in order to keep the roots intact, where the DNA is concentrated [69]. Nevertheless, it should be noted that the amount of DNA in each hair (e.g., on average 200 ng of DNA compared to 10 ng in fallen hairs) depends on the anatomic place of collection (e.g., head, beard, or pubis) and varies between individuals [68]. Moreover, the hair melanin is an important inhibitor of PCR DNA amplification, and therefore roots are preferable. Hair chemical treatments may also decrease the DNA yields.

### 5.3. Labeling, Packaging, and Storage of Evidence

**Figure 1:**
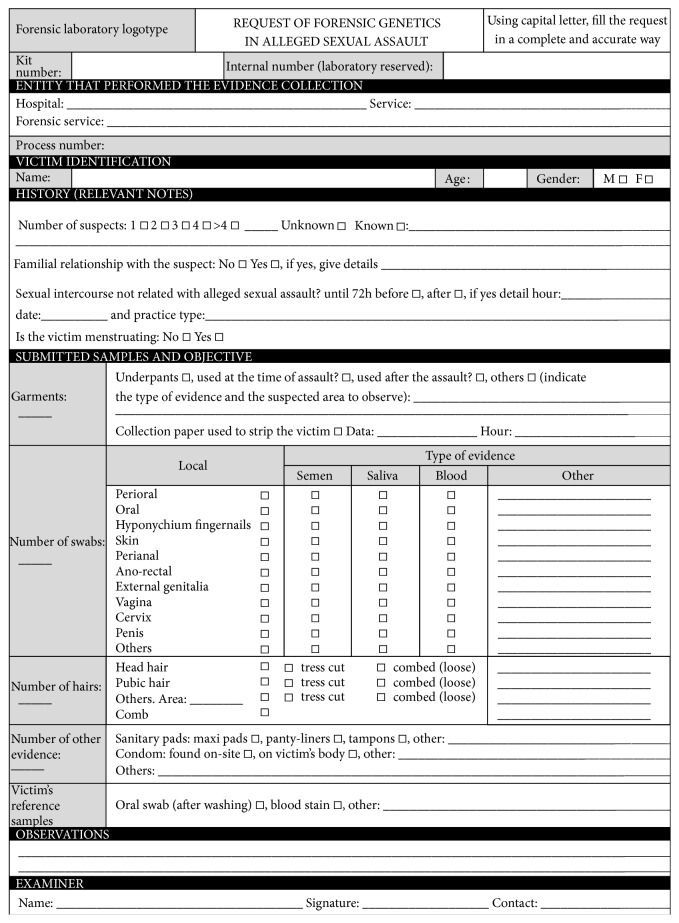
Forensic Genetics request form used for sexual assault cases.

Evidence must be properly labeled and packaged, in order to ensure that evidence is not lost, damaged, or contaminated until handled by the laboratory personnel and to guarantee that reliable results and the chain of custody compliance to evidence be admissible in a court of law. Therefore, all professionals involved need to respect strict procedures [7, 48, 70, 71] that will be briefly discussed:

- Each evidence must be dried before packaging. If a damp swab or other biological evidence is placed in a plastic or glass container, it will create a favorable environment to the development of bacteria and fungus, thus accelerating the degradation of DNA. If drying is not possible, evidence should be frozen (e.g., hygienic pads or tampons with blood).
- Paper foldable racks, packages, or bags are preferred for biological evidence instead of plastic or glass containers, since paper allows remaining humidity to evaporate. Plastic or glass containers could be used to package physical evidence.
- For debris such as hairs, leaves, and fibers, a bindle can be used and then put into a paper package (double packaged).
- All evidence should be individually sealed.
- Self-sealing envelopes or suitable adhesive should be used. If the glue is to be moistened, this procedure should be accomplished with tap water or soaked gauze and not saliva, to prevent the contamination of DNA.
- Evidence should be clearly labeled with at least case number, victim's examiner's names, collection date and time, sample type, evidence number, and the location from which the evidence was collected on the victim's body. Ideally a barcode should be used.
- The request forms should be carefully filled. In Figure 1 we present an example of a request form for Forensic Genetics in case of sexual assault.
- The examiner should put the request form together with samples inside the respective kit before sealing it.
- Packages should not be stapled and must be signed across the seal in order to detect possible fraud. A biohazard label must be affixed to the package if needed.
- Each kit should be kept in a suitable and secure place with adequate environmental conditions (e.g., DNA samples are either stored in a refrigerator at 4°C or a freezer at −20°C to reduce microorganisms' growth rate and to avoid DNA degradation).
- The kit should be shipped to the laboratory as soon as possible.
- Regardless of the transportation means, it is important to ensure that samples are not exchanged/switched from time of collection to receipt in the laboratory.
- All individuals handling samples must sign appropriate chain of custody report to track documentation regarding date, time, and names.

## 6. Discussion

Biological evidence is very important, especially in ASA, since it may prove the existence of sexual contact and lead to the identification of a perpetrator. Knowing and respecting the best practices of evidence management is essential to ensure that evidence (sometimes found in low quantities) is not lost, destroyed, or contaminated and to guarantee reliable results and the admissibility of evidence in the court of law. Carelessness or ignorance of proper handling procedures can result in a sample unsuitable for analysis and in the acquittal of a perpetrator. With this work we intended to summarize and harmonize FME procedures with regard to evidence management for DNA analysis, specifically the selection, collection, packaging, storage, preservation, and transportation of the evidence to the laboratories. Knowing and respecting the good practices of evidence management is essential to ensure that it is not lost, destroyed, or contaminated and to guarantee reliable results and the admissibility of evidence in court of law. Carelessness or ignorance of proper handling procedures for biological evidence can result in an unfit sample for analysis and in the acquittal of the perpetrator. The victim is entitled to a fair judicial decision.

Finally, it is important to highlight all steps that any forensic medical expert should be aware of in the management of evidence for DNA analysis:

- Sexual assault history and the physical observation which should guide the examiner for evidence collection (e.g., victim's activities between the sexual contact and the examination, victim's gender and age, and type of evidence).
- Performing a proper collection, avoiding loss or contamination (specially cross-contamination).
- Drying under suitable conditions.
- Individualized packaging.
- Sealing of containers.
- Labeling and signing of packages.
- Correct and complete filling out of forms requesting laboratory analysis.
- Storing the evidence along with the request form into a kit box or appropriate envelope or bag, guaranteeing the adequate conditions of conservation and security.
- Sealing of kits.
- Labeling and signing kits; each kit must be assigned a number and must contain many labels printed with this number.
- Adequate environmental and secure storage of kits.
- Delivering to the forensic laboratory all kits and the sealed bag with clothing, accompanied with the chain of custody forms signed by all individuals.
